# Complete Heart Block: A Rare Complication of Takotsubo Syndrome

**DOI:** 10.1155/2019/2576373

**Published:** 2019-10-09

**Authors:** N. F. N. Sakul, Srijan Shrestha, Nikhita Balabbigari, Sapan Talati

**Affiliations:** ^1^Department of Internal Medicine, Overlook Medical Center, Summit, NJ 07901, USA; ^2^Department of Cardiology, Morristown Medical Center, Morristown, NJ 07960, USA

## Abstract

Takotsubo syndrome was believed to be a rare acute cardiac event until recently with takotsubo cardiomyopathy being its most commonly recognized and often the diagnostic feature. Its diagnosis is becoming increasingly common with varied clinical manifestations most of whom have favorable clinical outcomes, yet it can be associated with life-threatening complications. We report a case of takotsubo syndrome leading to complete heart block which is a unique complication of an otherwise self-resolving disease.

## 1. Introduction

Takotsubo syndrome as a cardiomyopathy is a distinct form of nonischemic cardiomyopathy characterized by abrupt onset transient regional dysfunction [1]. It is usually precipitated by an identifiable stressor and frequently presents with chest pain. The presence of arrhythmias is rare and the most common EKG abnormalities include ST segment elevation and T-wave inversions [2]. In recent years, some case reports have emerged regarding an uncommon association of AV blocks in takotsubo cardiomyopathy. While in this syndrome, the cardiomyopathy usually reverses itself, the AV blocks may not. This poses a therapeutic dilemma with respect to a possible need for intervention, such as pacemaker [3], for what was thought to be a relatively benign condition.

## 2. Case Presentation

**Figure 1:**
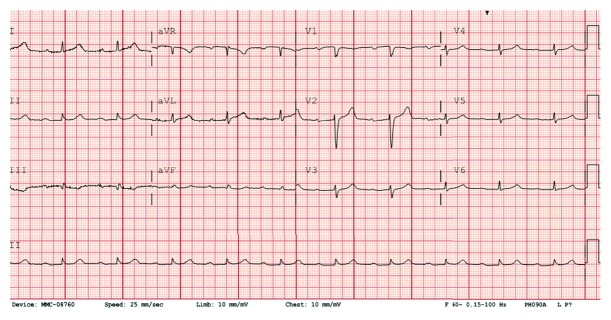
EKG on presentation showing sinus rhythm with prolonged PR interval.

This is a 73-year-old Caucasian female with history of hypertension and irritable bowel syndrome who presented to the hospital due to sudden onset retrosternal, squeezing, nonradiating chest heaviness during an episode of diarrhea. The pain was associated with minimal shortness of breath. She received aspirin 325 mg by EMS along with sublingual nitroglycerin. On examination in the emergency room, she had a blood pressure of 115/64 mm Hg, a regular heart rate of 67 beats per minute, a respiratory rate of 18 breaths per minute, and saturating 97% on room air. She had normal breath sounds and heart sounds on examination. She was started on nitroglycerin infusion, after which her chest heaviness partially improved but persisted. Initial EKG showed no acute changes but prolonged PR interval of 324 milliseconds (Figure 1).

The patient was known to have a history of prolonged PR interval noted on prior EKGs. Initial labs showed serum sodium 136 mmol/L, potassium 4.1 mmol/L, bicarbonate 22 mmol/L, chloride 102 mmol/L, BUN 42 mg/dL, creatinine 1 mg/dL, glucose 118 mg/dL, calcium 8.7 mg/dL, white cell count 7.3/nL, hemoglobin 12.1 g/dL, and platelet count 219/nL. Coagulation profile and hepatic function panel were also within normal limits. Initial troponin was 0.46 ng/dL which increased to 3.06 ng/dL after five hours. An initial diagnosis of NSTEMI was made, and the patient was started on a heparin infusion. Urgent coronary catheterization was organized.

**Figure 2:**
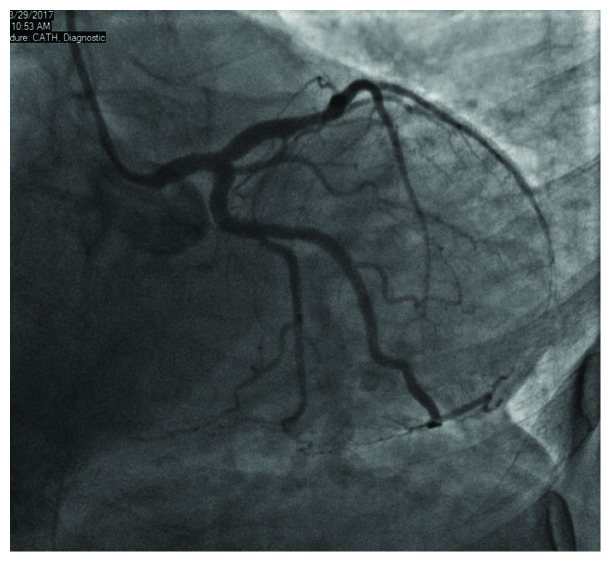
Coronary angiogram showing LAD caudal view.

**Figure 3:**
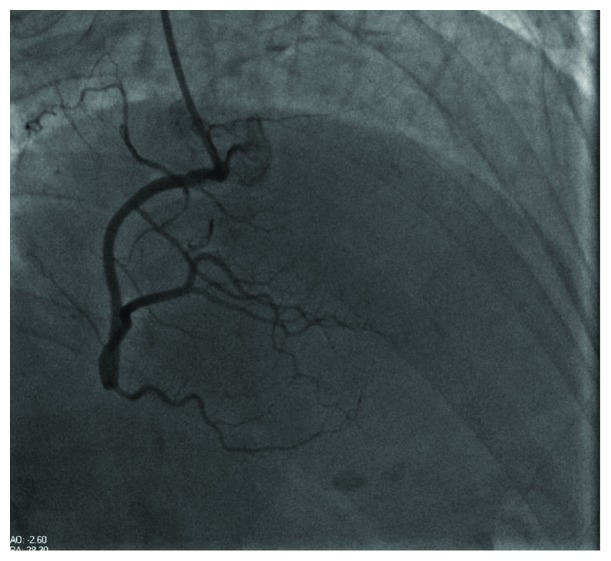
Coronary angiogram showing a view of the right coronary artery.

**Figure 4:**
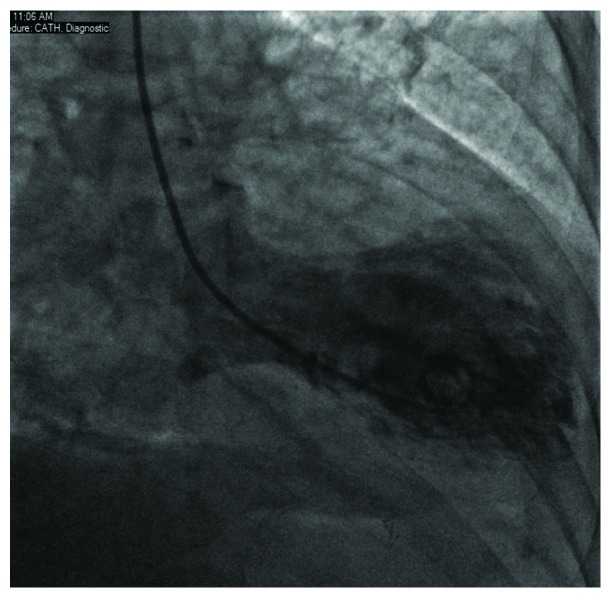
Left ventriculogram in systole.

**Figure 5:**
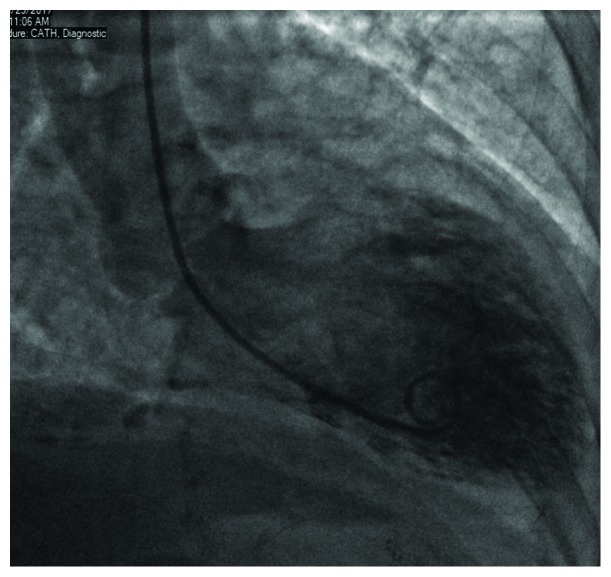
Left ventriculogram in diastole.

**Figure 6:**
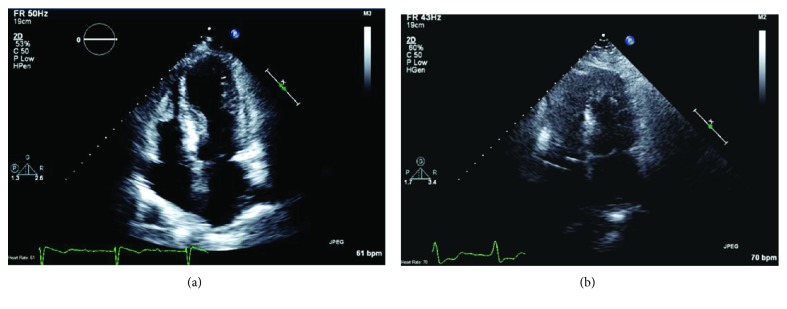
Apical 4 chamber ECHO view (a) on presentation suggestive of takotsubo cardiomyopathy followed by repeat apical 4 chamber view (b) two months later showing recovery of previously noted wall motion abnormalities.

**Figure 7:**
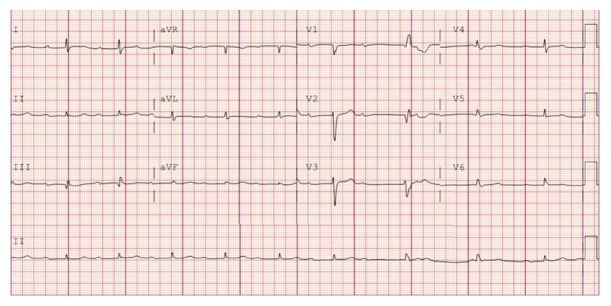
EKG showing Mobitz type 1 AV block.

**Figure 8:**
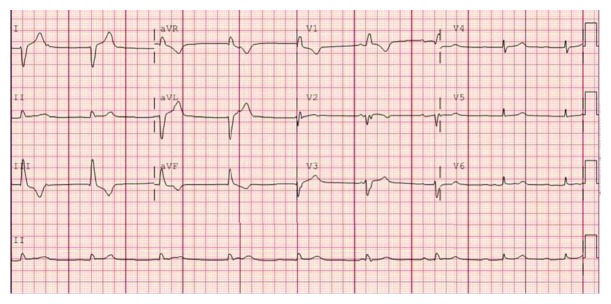
EKG showing third degree complete AV block.

Coronary angiogram demonstrated a nonobstructive coronary artery disease (Figures 2 and 3) with a ventriculogram showing apical ballooning and ejection fraction of 40% suggestive of takotsubo cardiomyopathy (Figures 4 and 5). The patient was admitted to the CCU, and a transthoracic echo was done and consistent with the findings of coronary catheterization: it showed an ejection fraction of 45% with hyperdynamic inferobasal and anterior basal walls with apical ballooning indicative of takotsubo cardiomyopathy (Figure 6). She was diagnosed as having takotsubo syndrome, and her heparin infusion was discontinued. Her hospitalization was complicated by Mobitz 1 AV block (Figure 7), wide complex escape rhythms, and subsequent complete heart block (Figure 8). Because of her baseline prolonged PR interval and very high projected pacing burden, biventricular pacer was inserted. The patient remained hemodynamically stable and was discharged home after a couple of days.

A repeat echocardiogram was done outpatient, two months after hospitalization, and showed improvement: she had an ejection fraction of 55-60% with resolution of the apical ballooning and hyperdynamic wall motion changes, which were initially noted during hospitalization (Figure 6). Pacemaker interrogation revealed 100% ventricular pacing and continues to show 100% ventricular pacing even twenty months after hospital discharge. This signifies the persistence and chronicity of the AV block even after her hospitalization and after the resolution of her cardiomyopathy.

## 3. Discussion

Takotsubo syndrome is deemed clinically benign with most characteristic features reversing with supportive therapy within months. The wall motion abnormalities characteristic of takotsubo cardiomyopathy usually reverse completely. High-degree AV blocks and complete heart block accompanying takotsubo cardiomyopathy are a rare phenomenon [4]. However, unlike some other features associated with takotsubo cardiomyopathy, AV blocks may persist despite optimal medical therapy and complete clinical recovery from other features. This may indicate that the recovery of the contractile system in takotsubo cardiomyopathy is faster than the recovery of the cardiac conduction system. Baranchuk et al. demonstrated a case in which high-degree AV block was present even after one year of initial takotsubo cardiomyopathy event but eventually resolved after 2 years of follow-up [5]. In our patient, pacemaker interrogation after 20 months of inciting event still suggested persistence of high AV block. The pathogenesis of takotsubo syndrome is not well understood, but catecholamine excess has been postulated as being central to the cause [2]. Some studies have even demonstrated higher circulating levels of epinephrine and norepinephrine levels in patients with takotsubo syndrome compared to patients diagnosed with myocardial infarction. Although conventional coronary angiogram does not show any obstructive lesions, some researchers suggest diffuse spasm of small branches of coronary arteries which may cause ischemia as an alternate mechanism of takotsubo cardiomyopathy [6, 7]. The latter mechanism may also explain the presence of AV conduction defects in takotsubo cardiomyopathy. There have been case reports in which AV block was shown to be the cause of takotsubo Syndrome, and the persistence of AV block in our patient despite improvement in her ventricular function raises the possibility of AV block with its subsequent symptoms as physical trigger for takotsubo syndrome. The absence of involvement of the basal segment of the left ventricle which is adjacent to the AV node also raises doubts about takotsubo syndrome causing AV block in our patient. But our patient was diagnosed as having takotsubo syndrome almost a day before continuous telemetry revealed her to be developing Mobitz type 1 block and ultimately complete heart block thus ruling out AV block as the cause of takotsubo syndrome in our patient.

## 4. Conclusion

Despite increasing knowledge and awareness of the historically enigmatic takotsubo syndrome, it is still believed to be a relatively benign and self-resolving entity. The recognition of AV conduction defects along with conventional and more common features is important as they may not resolve along with other features and may need intervention such as pacemaker implantation. Such situations are under recognized but are potentially life threatening and need timely management for patient safety.

## Supplementary Materials

Supplementary Materials This is a four-chamber apical view on a transthoracic echocardiogram of the patient during her index hospitalization which shows the hyperdynamic inferobasal and anterobasal walls with apical ballooning consistent with Takotsubo cardiomyopathy. It also shows her EF to be around 40% with normal biatrial size, normal valvular function, and no pericardial effusion.

## References

[1] Tsuchihashi K., Ueshima K., Uchida T. (2001). Transient left ventricular apical ballooning without coronary artery stenosis: a novel heart syndrome mimicking acute myocardial infarction. *Journal of the American College of Cardiology*.

[2] Brown K. H., Trohman R. G., Madias C. (2015). Arrhythmias in takotsubo cardiomyopathy. *Cardiac Electrophysiology Clinics*.

[3] Madias J. E. (2015). Delving into the issue of life-threatening arrhythmias in patients with takotsubo syndrome. *International Journal of Cardiology*.

[4] Syed F. F., Asirvatham S. J., Francis J. (2011). Arrhythmia occurrence with takotsubo cardiomyopathy: a literature review. *Europace*.

[5] Baranchuk A., Nault M., Redfearn D., Bally K., Simpson C. (2007). A novel “proarrhythmic” disease: takotsubo cardiomyopathy. *Journal of Electrocardiology*.

[6] Dote K., Sato H., Tateishi H., Uchida T., Ishihara M. (1991). Myocardial stunning due to simultaneous multivessel coronary spasms: a review of 5 cases. *Journal of Cardiology*.

[7] Kurisu S., Sato H., Kawagoe T. (2002). Tako-tsubo-like left ventricular dysfunction with ST-segment elevation: a novel cardiac syndrome mimicking acute myocardial infarction. *American Heart Journal*.

